# Hong Kong Hospital Authority resource efficiency evaluation: Via a novel DEA-Malmquist model and Tobit regression model

**DOI:** 10.1371/journal.pone.0184211

**Published:** 2017-09-08

**Authors:** Hainan Guo, Yang Zhao, Tie Niu, Kwok-Leung Tsui

**Affiliations:** 1 Research Institute of Business Analytics & Supply Chain Management, College of Management, Shenzhen University, Shenzhen, China; 2 Department of Systems Engineering and Engineering Management, City University of Hong Kong, Kowloon, Hong Kong; 3 Centre for Systems Informatics Engineering, City University of Hong Kong, Kowloon, Hong Kong; 4 Information Center, Hospital Affiliated with Dalian Medical University, Liaoning, China; University of Hong Kong, HONG KONG

## Abstract

The Hospital Authority (HA) is a statutory body managing all the public hospitals and institutes in Hong Kong (HK). In recent decades, Hong Kong Hospital Authority (HKHA) has been making efforts to improve the healthcare services, but there still exist some problems like unfair resource allocation and poor management, as reported by the Hong Kong medical legislative committee. One critical consequence of these problems is low healthcare efficiency of hospitals, leading to low satisfaction among patients. Moreover, HKHA also suffers from the conflict between limited resource and growing demand. An effective evaluation of HA is important for resource planning and healthcare decision making. In this paper, we propose a two-phase method to evaluate HA efficiency for reducing healthcare expenditure and improving healthcare service. Specifically, in Phase I, we measure the HKHA efficiency changes from 2000 to 2013 by applying a novel DEA-Malmquist index with undesirable factors. In Phase II, we further explore the impact of some exogenous factors (e.g., population density) on HKHA efficiency by Tobit regression model. Empirical results show that there are significant differences between the efficiencies of different hospitals and clusters. In particular, it is found that the public hospital serving in a richer district has a relatively lower efficiency. To a certain extent, this reflects the socioeconomic reality in HK that people with better economic condition prefers receiving higher quality service from the private hospitals.

## Introduction

The Hospital Authority (HA) is a statutory body established under the Hospital Authority Ordinance in 1990, which manages all the public hospitals and institutes in Hong Kong (HK). The strategic priorities of Hong Kong Hospital Authority (HKHA) are to (i) allay staff shortage and high turnover; (ii) better manage growing service demand; (iii) ensure service quality and safety; (iv) enhance partnership with patients and community; (v) ensure adequate resources to meet the service demand; and (vi) enhance the corporate governance.

HKHA has built a world-class public healthcare infrastructure that handles around 90% of secondary and tertiary medical needs in HK. Although HKHA has been providing excellent and affordable public healthcare to citizens, it suffers from some problems like long waiting time and low service quality. In order to overcome these limitations, it is important for HA policy makers to evaluate the healthcare efficiency of hospitals, locate the bottlenecks within public healthcare service system, and establish countermeasures accordingly [1, 2].

Efficiency measurement is an effective way to evaluate the performance of target configuration in healthcare. Since the goals of healthcare services are always multiple, conflicting, intangible, and complex, it is difficult and intractable to set up an overall measurement of performance. Regarding the multiple inputs and outputs, Data Envelopment Analysis (DEA) has been recognized as an effective nonparametric model to assess the relative efficiencies of a set of decision-making units (DMUs) [3]. Particularly, in recent decades, DEA has been widely applied to improve the productivity of healthcare-related fields in many countries [4–7], such as the United States [8], Japan [9], China [10] and India [11]. As discussed in [12], DEA is more suitable for managerial decision-making, which makes it the best choice to evaluate the efficiencies of HKHA hospitals.

In DEA model, efficiency score is closely related to the quantities of inputs and outputs. An ideal scenario with the highest efficiency is producing the most outputs (e.g. the number of treated patients) using the least inputs (e.g. healthcare expenditure); and such inputs and outputs are called desirable factors. However, the maximum quantities of desirable outputs are usually bounded, given the limited desirable inputs. The reception of the patients beyond the hospital capacity will generate a congestion problem, even resulting in some unexpected mortality. A high patient mortality rate not only reflects a poor medical quality, but also may cause a dispute. Thus, the mortality rate of patient, as an undesirable output, is expected to be lower. The measurement of hospital efficiency might be biased if the undesirable factors are ignored.

In order to integrate undesirable factors into modeling, one straightforward idea is applying inversion transformation on the value of undesirable factors [13]. However, the transformed values are usually negative, making it difficult for DEA modeling. To circumvent this difficulty, one solution is adding a constant to the transformed undesirable factors to keep them positive [14–16]. However, the determination of this constant has a significant impact on DMU ranking and classification. Another widely used transformation is the reciprocal transformation [17–19], but the computation complexity of this nonlinear transformation is much higher. Färe and Grosskopf [20, 21] propose an alternative approach which handles the undesirable factors by using a directional distance function. However, the form of direction vector could affect the DMU ranking. Thus, it is a critical issue to design an appropriate transformation function. In some cases, an improper transformation may generate the inverse result as it is expected. To avoid the transformation problem and preserve the original data, Liu and Sharp [22] treat the undesirable inputs as desirable outputs and vice versa, based on the physical relationship between the input and output. It has been proved as an effective approach which considers computing the operational efficiency by maximizing the undesirable inputs and desirable outputs and minimizing the desirable inputs and undesirable outputs, simultaneously. This idea is further extended by [23], where a unified model with several different DEA approaches dealing with undesirable inputs or outputs is proposed. Besides, it is suggested that the weighted additive model [24] can be applied to handle the problem with undesirable inputs or outputs. In [25], a slacks-based measure (SBM) is proposed, in which the unit invariant, monotone, and reference-set dependent properties are analyzed in details. The proposed SBM model outperforms CCR, BCC and Russell model in efficiency evaluation, where both the slacks for the input and the output are calculated. In [26], a more generalized slacks-based DEA model with undesirable factors considering preference is proposed, which combines the advantages of all the factors stated above and makes several DEA models unified. The proposed method also outperforms some state-of-the-art DEA models on different databases of real applications.

In this paper, we focus on evaluating the healthcare efficiency of HKHA from 2000 to 2013 by applying the Malmquist index [27] based on the updated DEA model developed by [26]. The Malmquist index can be well applied to track the specific position corresponding to each hospital and to examine changes in productivity and quality [28–31]. The empirical results reveal that the HA hospitals with low efficiencies may be caused by unfair resource allocation and inefficient resource utilization.

Additionally, in the context of healthcare measurement, we have found only sparse literature considering the *exogenous* factors [32, 33], as they are usually beyond the managers’ control. For example, population density and median monthly income could not be reduced in the same way as the healthcare expenditures. In this study, we apply the Tobit regression model to explore the impact of these exogenous factors. The experimental results reflect an interesting phenomenon that the HA hospitals serving in the richer clusters have the comparatively lower efficiency values in HK, since people with higher income prefer to accepting treatment from the private hospitals due to the better service quality.

## Methodology

DEA has been recognized as an effective nonparametric model to assess the relative efficiencies of a set of DMUs which consume multiple inputs to produce multiple outputs [34]. DEA models evaluate the relative efficiency of DMUs by creating a production frontier using the best practice of observed data. A DMU is to be rated as fully efficient on the basis of available evidence if and only if the performances of other DMUs does not show that some of its inputs or outputs can be improved without worsening some of its other inputs or outputs. After more than thirty-five years of development, research related to DEA is still growing at a very fast rate. Liu et al. [35] has summarized that during the period 2010 to 2014, around 2,000 more DEA-related papers have been published, in addition to the existing 4,500 collections before 2010 as reported by Liu et al. [36].

In this section, we first introduce a novel generalized and slacks-based non-oriented DEA model, and then, combine it with the Malmquist index.

### GSBUP-DEA

GSBUP (Generalized Slacks-Based DEA model with Undesirable factors considering Preference) [26] is a non-oriented DEA model, in which the undesirable inputs are regarded as the desirable outputs and vice versa, according to the physical relationship between them [10]. The preferences of different factors for DMUs are integrated into the model in terms of the multiplicative weights. The combination of both orientations is proposed to avoid dealing with different results calculated in an input-oriented or an output-oriented manner under variable return scale environment.

Assume that there are *n* DMUs to be evaluated, and each DMU_*j*_(*j* = 1, 2, …, *n*) is assumed to consume *m* desirable inputs $x_{ij}^{D}\left(i=1,2,\ldots ,m\right)$ and *q* undesirable inputs $x_{lj}^{I}\left(l=1,2,\ldots ,q\right)$ to produce *s* desirable outputs $y_{rj}^{G}\left(r=1,2,\ldots ,s\right)$ and *k* undesirable outputs $y_{hj}^{B}\left(h=1,2,\ldots ,k\right)$. Let $x_{j}^{D}=\left(x_{1j}^{D},\ldots ,x_{mj}^{D}\right)$, $x_{j}^{I}=\left(x_{1j}^{I},\ldots ,x_{qj}^{I}\right)$, $y_{j}^{G}=\left(y_{1j}^{G},\ldots ,y_{sj}^{G}\right)$ and $y_{j}^{B}=\left(y_{1j}^{B},\ldots ,y_{kj}^{B}\right)$ be the observed input and output vectors of DMU_*j*_, respectively. Note that all components of these vectors have non-negative values and each DMU has at least one strictly positive input and output. If the vector (***x***^*D*^, ***y***^*G*^, ***x***^*I*^, ***y***^*B*^) indicates a production plan, then the Production Possibility Set (PPS) of GSBUP-DEA is defined as follows:

$$\begin{array}{ccc} T & = & \{\left(x^{D},y^{G},x^{I},y^{B}\right)|x^{D}\geq \sum_{j}\lambda_{j}x_{j}^{D},\;y^{G}\leq \sum_{j}\lambda_{j}y_{j}^{G},\\ & & x^{I}\leq \sum_{j}\lambda_{j}x_{j}^{I},\;y^{B}\geq \sum_{j}\lambda_{j}y_{j}^{B},\;\sum_{j}\lambda_{j}\in \Gamma ,\;\lambda_{j}\in R_{+}^{n}\} \end{array}$$ (1)

where $R_{+}^{n}=\left\{\lambda_{j}\geq 0,\;j=1,2,\ldots ,n\right\}$ and Γ ∈ {IRS, DRS, CRS} with $\text{IRS}=\{\sum_{j}\lambda_{j}<1\}$, $\text{DRS}=\{\sum_{j}\lambda_{j}>1\}$ and $\text{CRS}=\{\sum_{j}\lambda_{j}=1\}$. A DMU_0_ is efficient if there is no vector $(x_{0}^{D},y_{0}^{G},x_{0}^{I},y_{0}^{B})\in T$ let $x_{0}^{D}\geq x^{D}$, $y_{0}^{G}\leq y^{G}$, $x_{0}^{I}\leq x^{I}$, and $y_{0}^{B}\geq y^{B}$ with at least one strict inequality.

For a ${\text{DMU}}_{0}=\left(x_{0}^{D},y_{0}^{G},x_{0}^{I},y_{0}^{B}\right)$, the inequalities in Eq (1) can be transformed into equalities by introducing the slacks as follows:

$$\begin{array}{cccc} & \sum_{j}\lambda_{j}x_{j}^{D}=x_{0}^{D}-s^{D-} & \; & \sum_{j}\lambda_{j}x_{j}^{I}=x_{0}^{I}+s^{I+}\\ & \sum_{j}\lambda_{j}y_{j}^{G}=y_{0}^{G}+s^{G+} & \; & \sum_{j}\lambda_{j}y_{j}^{B}=y_{0}^{B}-s^{B-} \end{array}$$ (2)

where $s^{D-}=\left(s_{1}^{D-},s_{2}^{D-},\ldots ,s_{m}^{D-}\right)$ ∈ $R_{+}^{m}$, $s^{G+}=\left(s_{1}^{G+},s_{2}^{G+},\ldots ,s_{s}^{G+}\right)$ ∈ $R_{+}^{s}$, $s^{I+}=\left(s_{1}^{I+},s_{2}^{I+},\ldots ,s_{q}^{I+}\right)$ ∈ $R_{+}^{q}$, and $s^{B-}=\left(s_{1}^{B-},s_{2}^{B-},\ldots ,s_{k}^{B-}\right)$ ∈ $R_{+}^{k}$ are defined as desirable input (DI) excess, desirable output (DO) shortfall, undesirable input (UI) shortfall, and undesirable output (UO) excess, respectively.

Let

$$\begin{array}{cccc} & s^{D-}=\alpha^{{}^{\prime }}\cdot x_{0}^{D} & \; & s^{G+}=\beta^{{}^{\prime }}\cdot y_{0}^{G}\\ & s^{I+}=\gamma^{{}^{\prime }}\cdot x_{0}^{I} & \; & s^{B-}=\theta^{{}^{\prime }}\cdot y_{0}^{B} \end{array}$$

Thus, Eq (2) can be transformed as below:

$$\begin{array}{cccc} & \sum_{j}\lambda_{j}x_{j}^{D}=\left(1-\alpha^{{}^{\prime }}\right)\cdot x_{0}^{D} & \; & \sum_{j}\lambda_{j}x_{j}^{I}=\left(1+\gamma^{{}^{\prime }}\right)\cdot x_{0}^{I}\\ & \sum_{j}\lambda_{j}y_{j}^{G}=\left(1+\beta^{{}^{\prime }}\right)\cdot y_{0}^{G} & \; & \sum_{j}\lambda_{j}y_{j}^{B}=\left(1-\theta^{{}^{\prime }}\right)\cdot y_{0}^{B} \end{array}$$ (3)

where “⋅” denotes the element-wise multiplication between vectors, $\alpha =1-\alpha^{{}^{\prime }}=\left(\alpha_{1},\alpha_{2},\ldots ,\alpha_{m}\right)\in R_{+}^{m}$, $\beta =1+\beta^{{}^{\prime }}=\left(\beta_{1},\beta_{2},\ldots ,\beta_{s}\right)\in R_{+}^{s}$, $\gamma =1+\gamma^{{}^{\prime }}=\left(\gamma_{1},\gamma_{2},\ldots ,\gamma_{q}\right)\in R_{+}^{q}$, and $\theta =1-\theta^{{}^{\prime }}=\left(\theta_{1},\theta_{2},\ldots ,\theta_{k}\right)\in R_{+}^{k}$.

In order to take DI, DO, UI, and UO into account simultaneously, and to integrate efficiency and slacks into a scalar measure, the GSBUP model is developed by applying the transformation approach mentioned above:

**[GSBUP]**

$$\begin{array}{cc} & \min\;\;\delta_{0}=\frac{\sum_{i=1}^{m}\omega_{i}\alpha_{i}+\sum_{h=1}^{k}\nu_{h}\theta_{h}}{\sum_{l=1}^{q}\sigma_{l}\gamma_{l}+\sum_{r=1}^{s}\mu_{r}\beta_{r}}\\ & s.t.\;\left\{ \begin{array}{c} \sum_{j=1}^{n}x_{ij}^{D}\lambda_{j}=\alpha_{i}x_{i0}^{D}\;\left(i=1,2,\ldots ,m\right)\\ \sum_{j=1}^{n}x_{lj}^{I}\lambda_{j}=\gamma_{l}x_{l0}^{I}\;\left(l=1,2,\ldots ,q\right)\\ \sum_{j=1}^{n}y_{rj}^{G}\lambda_{j}=\beta_{r}y_{r0}^{G}\;\left(r=1,2,\ldots ,s\right)\\ \sum_{j=1}^{n}y_{hj}^{B}\lambda_{j}=\theta_{h}y_{h0}^{B}\;\left(h=1,2,\ldots ,k\right)\\ \sum_{j=1}^{n}\lambda_{j}\in \Gamma \\ \lambda_{j}\geq 0,\;0\leq \alpha_{i}\leq 1,\;\gamma_{l}\geq 1,\;\beta_{r}\geq 1,\;0\leq \theta_{h}\leq 1\;\left(\forall j,i,l,r,h\right) \end{array}\right. \end{array}$$ (4)

where *ω*_*i*_, *μ*_*r*_, *σ*_*l*_, and *ν*_*h*_ (∀*i*, *r*, *l*, *h*) denote the preferences of DI, DO, UI, and UO, respectively, where $\sum_{i=1}^{m}\omega_{i}+\sum_{h=1}^{k}\nu_{h}=1$ and $\sum_{l=1}^{q}\sigma_{l}+\sum_{r=1}^{s}\mu_{r}=1$. The decision variables are *α*_*i*_, *β*_*r*_, *γ*_*l*_, and *θ*_*h*_ (∀*i*, *r*, *l*, *h*), which also contain the slacks of all the factors.

In order to evaluate the relative efficiency of ${\text{DMU}}_{0}=\left(x_{0}^{D},y_{0}^{G},x_{0}^{I},y_{0}^{B}\right)$, we solve the above **[GSBUP]** model. This process is repeated times for 0 = (1, 2, …, *n*). Once GSBUP identifies the efficient frontier, it can improve the performance of inefficient DMUs by either increasing the current DO (or UI) levels or decreasing the current DI (or UO) levels. Thus, the objective of **[GSBUP]** is to minimize the ratio of the weighted efficiency summation of the DIs and UOs to that of the DOs and UIs. The details of the linear programming (LP) problem of GSBUP is given in Appendix A

We use two definitions and one theorem to further illustrate the properties of GSBUP.

**Definition 1.** (GSBUP-Efficient). *A* ${\text{DMU}}_{0}=\left(x_{0}^{D},y_{0}^{G},x_{0}^{I},y_{0}^{B}\right)$ *is called GSBUP-efficient if and only if when the optimal solution* (α*, β*, γ*, θ*) = (**1**, **1**, **1**, **1**) *holds. This is equivalent to* (***s***^*D*−∗^, ***s***^*G*+∗^, ***s***^*I*+∗^, ***s***^*B*−∗^) = (**0**, **0**, **0**, **0**). *Otherwise, the* DMU_0_ *is GSBUP-inefficient*.

**Definition 2.** (Projection). *Using an optimal solution* (λ*, α*, β*, γ*, θ*), *we define a projection of GSBUP* ${\hat{\text{DMU}}}_{0}=\left({\hat{x}}_{0}^{D},{\hat{y}}_{0}^{G},{\hat{x}}_{0}^{I},{\hat{y}}_{0}^{B}\right)$ *by*

$$\begin{array}{cccc} & {\hat{x}}_{0}^{D}=\sum_{j}\lambda_{j}^{*}x_{j}^{D}=\alpha^{*}\cdot x_{0}^{D} & \; & {\hat{x}}_{0}^{I}=\sum_{j}\lambda_{j}^{*}x_{j}^{I}=\gamma^{*}\cdot x_{0}^{I}\\ & {\hat{y}}_{0}^{G}=\sum_{j}\lambda_{j}^{*}y_{j}^{G}=\beta^{*}\cdot y_{0}^{G} & \; & {\hat{y}}_{0}^{B}=\sum_{j}\lambda_{j}^{*}y_{j}^{B}=\theta^{*}\cdot y_{0}^{B} \end{array}$$ (5)

**Theorem 1.** *Though* DMU_0_ *is GSBUP-inefficient, its projected* ${\hat{\text{DMU}}}_{0}$ *is GSBUP-efficient relative to the original set of* *n* *DMUs*.

*proof*. See Appendix B.

### GSBUP-based malmquist productivity index

DEA-based Malmquist productivity index, developed by [27], provides an evaluation of productivity change over time. To describe the GSBUP-based Malmquist productivity method, let $(x_{0}^{D,t}$, $y_{0}^{G,t},x_{0}^{I,t},y_{0}^{B,t})$ denote the input and output levels for a DMU_*j*_ at any given point in time *t*. From time *t* to *t* + 1, DMU_0_’s technical efficiency and empirical production frontier may change. The calculation of Malmquist index requires two single period and two mixed period evaluations according to GSBUP-DEA Model (4), shown as follows:

1. Comparing $(x_{0}^{D,t}$, $y_{0}^{G,t},x_{0}^{I,t},y_{0}^{B,t})$ to the frontier at time *t*, i.e., calculating $\delta_{0}^{t}\left(x_{0}^{D,t},y_{0}^{G,t},x_{0}^{I,t},y_{0}^{B,t}\right)$;
2. Comparing $\left(x_{0}^{D,t+1},y_{0}^{G,t+1},x_{0}^{I,t+1},y_{0}^{B,t+1}\right)$ to the frontier at time *t* + 1, i.e., calculating $\delta_{0}^{t+1}\left(x_{0}^{D,t+1},y_{0}^{G,t+1},x_{0}^{I,t+1},y_{0}^{B,t+1}\right)$;
3. Comparing $\left(x_{0}^{D,t},y_{0}^{G,t},x_{0}^{I,t},y_{0}^{B,t}\right)$ to the frontier at time *t* + 1, i.e., calculating $\delta_{0}^{t+1}\left(x_{0}^{D,t},y_{0}^{G,t},x_{0}^{I,t},y_{0}^{B,t}\right)$;
4. Comparing $\left(x_{0}^{D,t+1},y_{0}^{G,t+1},x_{0}^{I,t+1},y_{0}^{B,t+1}\right)$ to the frontier at time *t*, i.e., calculating $\delta_{0}^{t}\left(x_{0}^{D,t+1},y_{0}^{G,t+1},x_{0}^{I,t+1},y_{0}^{B,t+1}\right)$.

Then, the Malmquist productivity index is defined as follows (the calculation of each period is shown in Appendix C):

$$\begin{array}{c} {\text{M}}_{0}={\left[\frac{\delta_{0}^{t}\left(x_{0}^{D,t},y_{0}^{G,t},x_{0}^{I,t},y_{0}^{B,t}\right)}{\delta_{0}^{t}\left(x_{0}^{D,t+1},y_{0}^{G,t+1},x_{0}^{I,t+1},y_{0}^{B,t+1}\right)}\times \frac{\delta_{0}^{t+1}\left(x_{0}^{D,t},y_{0}^{G,t},x_{0}^{I,t},y_{0}^{B,t}\right)}{\delta_{0}^{t+1}\left(x_{0}^{D,t+1},y_{0}^{G,t+1},x_{0}^{I,t+1},y_{0}^{B,t+1}\right)}\right]}^{1/2} \end{array}$$ (6)

where M_0_ measures the productivity change between periods *t* and *t* + 1. Productivity declines if M_0_ > 1, remains the same if M_0_ = 1 and improves if M_0_ < 1. The M_0_ can be divided into two components, the change of technical efficiency (TEC) and the movement of the frontier (FS) in terms of a specific DMU_0_:

$$\begin{array}{c} {\text{M}}_{0}={\text{TEC}}_{0}\cdot {\text{FS}}_{0} \end{array}$$

where

$$\begin{array}{c} {\text{TEC}}_{0}=\frac{\delta_{0}^{t}\left(x_{0}^{D,t},y_{0}^{G,t},x_{0}^{I,t},y_{0}^{B,t}\right)}{\delta_{0}^{t+1}\left(x_{0}^{D,t+1},y_{0}^{G,t+1},x_{0}^{I,t+1},y_{0}^{B,t+1}\right)} \end{array}$$

and

$$\begin{array}{c} {\text{FS}}_{0}={\left[\frac{\delta_{0}^{t+1}\left(x_{0}^{D,t+1},y_{0}^{G,t+1},x_{0}^{I,t+1},y_{0}^{B,t+1}\right)}{\delta_{0}^{t}\left(x_{0}^{D,t+1},y_{0}^{G,t+1},x_{0}^{I,t+1},y_{0}^{B,t+1}\right)}\times \frac{\delta_{0}^{t+1}\left(x_{0}^{D,t},y_{0}^{G,t},x_{0}^{I,t},y_{0}^{B,t}\right)}{\delta_{0}^{t}\left(x_{0}^{D,t},y_{0}^{G,t},x_{0}^{I,t},y_{0}^{B,t}\right)}\right]}^{1/2} \end{array}$$

Similarly, TEC_0_ > 1, TEC_0_ = 1 and TEC_0_ < 1 indicates that technical efficiency declines, remains unchanged and improves, respectively. And the value of FS_0_ > 1 indicates regress in the frontier technology; while FS_0_ < 1 means progress in the frontier technology; naturally, FS_0_ = 1 indicates no shift in the frontier technology.

## Experimental studies and analysis: HKHA efficiency

In this section, we first analyze the HKHA data, and then discuss the measurement results of hospital efficiency in HK from 2000 to 2013 by applying Malmquist index based on GSBUP-DEA. It is expected that the results are helpful for HKHA policy makers to control healthcare costs and improve healthcare efficiency while ensuing the service quality requirements.

### Description of DMUs

**Fig 1:**
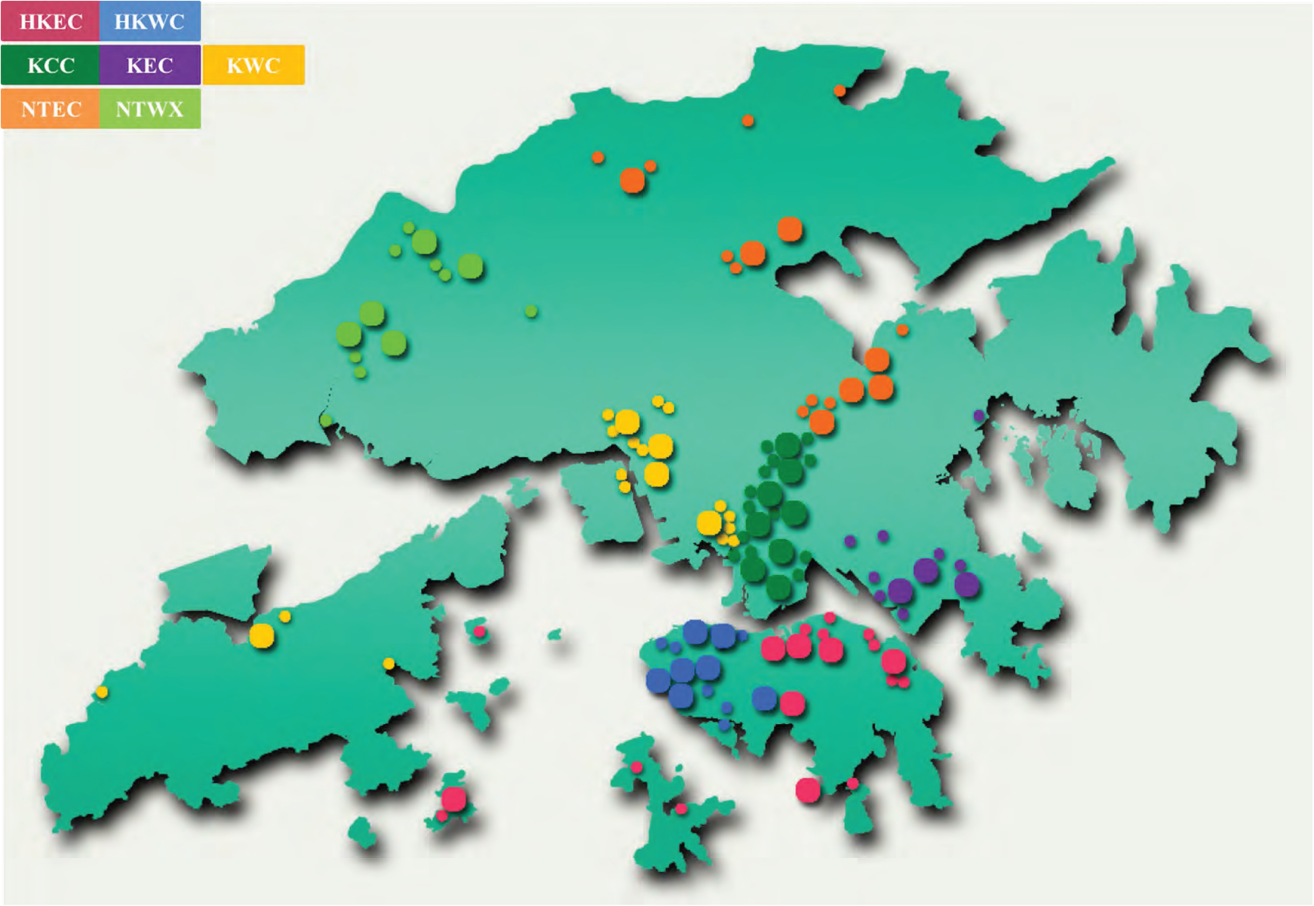
Distribution of clusters and hospitals.

To ensure patients in HK receive a continuum of high quality healthcare within the same area, HKHA divides the public hospitals into seven hospital clusters based on geographical locations, including Hong Kong East Cluster (HKEC), Hong Kong West Cluster (HKWC), Kowloon Central Cluster (KCC), Kowloon East Cluster (KEC), Kowloon West Cluster (KWC), New Territories East Cluster (NTEC), and New Territories West Cluster (NTWC), shown in Fig 1. Table 1 lists the specific hospitals in each cluster, which are also the DMUs evaluated in this study.

**Table 1:** Clusters of public hospitals in HK.

Cluster	Hospital
HKEC	Pamela Youde Nethersole Eastern Hospital
	Ruttonjee Hospital & Tang Shiu Kin Hospital
	St. John Hospital
	Tung Wah Eastern Hospital
HKWC	Queen Mary Hospital
	Tung Wah Hospital
KCC	Queen Elizabeth Hospital
KEC	Tseung Kwan O Hospital
	United Christian Hospital
KWC	Caritas Medical Centre
	Kwong Wah Hospital
	Princess Margaret Hospital
	Yan Chai Hospital
NTEC	Alice Ho Miu Ling Nethersole Hospital
	North District Hospital
	Prince of Wales Hospital
NTWC	Pok Oi Hospital
	Tuen Mun Hospital

### Input and output factors

Note that the evaluation of DEA performance highly depends on the selection of factors because the discriminatory power may be reduced when the dimensionality of the production space increases [37]. In this study, by reviewing the relevant literature, consulting with HK healthcare professionals and considering data availability and the parametric restrictions, two DIs (i.e., the number of full-time equivalent staff and the number of beds), one UI (i.e., in-patient discharge rate), DOs (i.e., total patient length of stay, total ED attendances and total outpatient attendances), and one UO (i.e., crude mortality rate) are selected for each DMU, as listed in Table 2.

**Table 2:** Factors selection.

Factor	Description	Unit
DI_1_	Number of Full-time Equivalent (FTE) Staff	1 person
DI_2_	Number of Beds	1 bed
UI_1_	In-patient Discharge Rate	%
DO_1_	Total In-patient (IP) and Day-patient (DP) Length of Stay	1000 days
DO_2_	Total ED attendances	1000 persons
DO_3_	Total Outpatient attendances	1000 persons
UO_1_	Crude Mortality Rate	‰

All the data are collected from the Hospital Authority Statistical Report [38]. As can be observed in Table 3, regarding the inputs of medical resources, there is a continuous increase of investment in both human resources and bed among hospitals. However, it is worth noting that except for the DIs, all the other factors (e.g., the utilization of bed, total patient attendances, and mortality rate) reach the peak values in 2003, especially the mortality rate, which is due to the outbreak of Severe Acute Respiratory Syndrome (SARS) [39]. In addition, the surge of patient attendances in 2013 is mainly caused by the winter influenza season and avian influenza (H7N9).

**Table 3:** Descriptive statistics for HKHA factors in each year.

Factor	Mean				
	2000	2003	2007	2010	2013
DI_1_	2112	2122	2278	2501	2747
DI_2_	998	999	1003	1021	1030
UI_1_	83	84	81	82	84
DO_1_	364	389	352	385	445
DO_2_	121	141	115	124	136
DO_3_	428	750	619	647	733
UO_1_	24	34	23	26	28

Table 4 shows the curde mortality rate of each HA hospital from 2000 to 2013. The crude mortality rate refers to the standardized hospital death rate covering inpatient and day patient deaths in HA hospitals during a year. The standardized death rate, as a standard statistical measure for temporal comparison, is calculated by applying the HA age-specific hospital death rate in the target year to the “standard” population in mid-2001. We observe that the Pok Oi Hospital, Ruttonjee Hospital & Tang Shiu Kin Hospital, Caritas Medical Centre, North District Hospital and Yan Chai Hospital rank top 5 regarding the value of crude mortality rate. The high crude mortality rates can be due to the facts that: (i) Pok Oi Hospital provides the elderly care for the people whose health condition and dependency level are beyond the coping capacity of care-and-attention home; (ii) Ruttonjee Hospital & Tang Shiu Kin Hospital, Caritas Medical Centre and Yan Chai Hospital are designated to provide the Geriatric Day Hospital; and (iii) North District Hospital has a relatively poorer technological level. Moreover, the heavy load on outpatient service may compete against the inpatient treatment, leading to a serious outcome of patient mortality. Therefore, it is necessary to consider this undesirable output to avoid overestimation.

**Table 4:** Curde mortality rate of HKHA, 2000-2013.

Hospital	2000	2003	2007	2010	2013	Average
Pamela Youde Nethersole Eastern	16.2	19.5	14.8	16.6	20.1	17.4
**Ruttonjee and Tang Shiu Kin**	34.8	53.7	32.4	50.7	54.0	45.1
St. John	9.0	9.3	9.4	5.5	24.1	11.5
Tung Wah Eastern	18.2	28.6	18.7	26.2	27.7	23.9
Queen Mary	15.4	17.4	10.3	13.3	16.1	14.5
Tung Wah	30.2	29.3	18.4	23.1	23.2	24.8
Queen Elizabeth	22.9	25.4	19.7	23.3	25.2	23.3
Tseung Kwan O	14.9	19.2	12.5	18.1	17.6	16.5
United Christian	18.4	24.6	17.6	19.0	23.6	20.6
**Caritas Medical Centre**	25.0	38.9	28.5	35.6	37.8	33.2
Kwong Wah	16.4	12.2	10.1	11.7	14.5	13.0
Princess Margaret	18.3	23.5	17.6	22.5	20.8	20.5
**Yan Chai**	22.9	35.8	25.2	31.4	34.2	29.9
Alice Ho Miu Ling Nethersole	11.0	16.2	12.8	14.4	15.9	14.1
**North District**	33.3	34.4	29.2	31.7	32.1	32.1
Prince of Wales	14.8	15.7	11.5	13.3	14.0	13.9
**Pok Oi**	82.3	182.5	98.6	78.4	81.8	104.7
Tuen Mun	21.4	25.2	21.6	25.0	22.0	23.0
**Average**	23.6	34.0	22.7	25.5	28.0	26.8

We use the data in 2013 as an example to illustrate the statistical characteristics of the specific input-output factors. It is observed in Table 5 that the median value of each factor is significantly different from the average value. Furthermore, the standard deviation values are large and the maximum can be 200 times larger than the minimum, indicating that the resource utilization levels and resource allocation of the clusters are seriously unbalanced. Thus, the analysis of HKHA hospital efficiency and the exploration of the influencing factors are of crucial importance for the policy makers.

**Table 5:** Descriptive statistical characteristics of input-output factors (year 2013).

Variable	Input factors			Output factors			
	DI_1_	DI_2_	UI_1_	DO_1_	DO_2_	DO_3_	UO_1_
Maximum	5870.2	1843.0	96.1	1381.2	228.9	1620.6	50.7
Minimum	113.3	87.0	61.6	14.3	4.2	39.7	5.5
Median	1995.0	991.5	82.6	368.1	138.0	647.3	20.6
Mean	2747.2	1029.9	84.2	445.0	125.7	732.7	21.6
SD	1805.2	563.5	8.5	326.7	61.8	477.3	10.4
Skewness	0.3	0.5	-0.7	1.3	-0.8	0.2	1.2
Kurtosis	-1.4	-1.5	1.2	2.2	0.4	-1.1	2.0

The correlation coefficients between all the input-output factors are given in Table 6. It is observed that there ia a significantly positive correlation between the DIs and DOs, satisfying the “isotonicity” production process that DOs do not decrease by increasing the DIs. Crucially, the mortality rate of patients has a significantly positive relationship with the DOs, which suggests that an increase in patient attendances is accompanied by an increase in patients’ death rate. In addition, the mortality rate of patients has a negative correlation with the amount of medical resources, which suggests that properly increasing the medical resources can relieve a poor service quality in a certain degree caused by resource limitation; while too many resources can increase the fiscal burden of government. Thus, it is a trade-off problem that how to determine the resource allocation.

**Table 6:** Pearson coefficient of input-output factors (year 2013).

Factor	DI_1_	DI_2_	UI_1_	DO_1_	DO_2_	DO_3_	UO_1_
DI_1_	1.000						
DI_2_	0.969	1.000					
UI_1_	0.633	0.666	1.000				
DO_1_	0.873	0.876	0.777	1.000			
DO_2_	0.795	0.784	0.844	0.729	1.000		
DO_3_	0.962	0.920	0.693	0.873	0.855	1.000	
UO_1_	-0.230	-0.143	0.852	0.857	0.747	0.879	1.000

### Experimental results

#### Evaluation of GSBUP-DEA model

We evaluate the resource efficiency of HKHA in each year by applying the LP of GSBUP-DEA Model (8). The efficiency scores for all the hospitals during 2000-2013 are listed in Table 7. According to Definition 1, the efficient DMUs’ $\delta_{0}^{*}$ are equal to 1. Thus, Tung Wah Hospital (2003-2013), Tseung Kwan O Hospital (2007-2013), Caritas Medical Centre (2000), Alice Ho Miu Ling Nethersole Hospital (2000 and 2010) and Tuen Mun Hospital (2013) are the efficient hospitals in resource operation. However, it is shown in Table 7 that there are only 55% hospitals have higher efficiency values than the average, 0.870, which reveals the fact that there exists unfair resource allocation, inefficient resource utilization and poor management in HKHA. For example, Pok Oi Hospital has relatively lower efficiency in the earlier years, because it had no ED service before 2010. Queen Mary Hospital is regarded as one of the best hospitals in HK, but it faces the over-investment problem which lowers the efficiency value.

**Table 7:** Efficiency scores of HKHA, 2000-2013.

Hospital	2000	2003	2007	2010	2013	RTS (2013)
Pamela Youde Nethersole Eastern	0.974	0.942	0.963	0.897	0.760	DRS
Ruttonjee and Tang Shiu Kin	0.847	0.769	0.786	0.807	0.771	IRS
St. John	0.963	0.974	0.864	0.920	0.894	IRS
Tung Wah Eastern	0.931	0.885	0.893	0.942	0.931	IRS
Queen Mary	0.816	0.784	0.822	0.787	0.677	DRS
**Tung Wah**	0.997	1	1	1	1	CRS(IRS)
Queen Elizabeth	0.964	0.923	0.954	0.938	0.794	DRS
**Tseung Kwan O**	0.843	0.727	1	1	1	CRS(IRS)
United Christian	0.975	0.902	0.936	0.906	0.748	DRS
**Caritas Medical Centre**	1	0.985	0.958	0.983	0.912	DRS
Kwong Wah	0.578	0.846	0.835	0.812	0.723	DRS
Princess Margaret	0.791	0.729	0.813	0.772	0.754	DRS
Yan Chai	0.811	0.771	0.827	0.832	0.797	DRS
**Alice Ho Miu Ling Nethersole**	1	0.975	0.928	1	0.964	DRS
North District	0.894	0.827	0.860	0.830	0.737	IRS
Prince of Wales	0.971	0.938	0.977	0.989	0.837	DRS
Pok Oi	0.564	0.535	0.480	0.900	0.989	IRS
**Tuen Mun**	0.950	0.930	0.968	0.971	1	CRS(DRS)
**Average**	0.859	0.858	0.881	0.905	0.849	
**No. of Efficiency**	2	1	2	3	3	
**No. of Greater than Average**	11	10	10	10	8	

Returns to scale (RTS) is the variation or change in productivity which describes the outcome from a proportionate increase of all the inputs. In DEA, an increasing return to scale (IRS) occurs when the output increases by a larger proportion than the inputs. Conversely, a decreasing return to scale (DRS) occurs when the proportion of output is less than the desired increased input during the production process. In Table 7, the last column displays the HA hospitals’ RTS in 2013. It reveals that if HKHA policy makers can properly increase the input of medical resources for the inefficient hospitals with IRS (i.e., Tung Wah Eastern Hospital and Pok Oi Hospital), these hospitals will achieve a higher service level than they expected. Notably, if HKHA policy makers continue to increase the hospital scale for the inefficient hospitals with DRS (i.e., Queen Mary Hospital and Prince of Wales Hospital), it will be an uneconomic production process and even introduces unnecessary fiscal burden to the government.

We take the year 2013 as an example, Table 8 shows the specific input-output factors, efficiency values and RTS status of each cluster. We find that KWC receives the most patient attendances; while KEC receives the least ones. In practice, HKHA allocates the medical resources for each cluster based on the patient attendances. Thus, HKHA should allocate the most medical resources for KWC and the least medical resources for KEC. However, this kind of method ignores the effect of medical case complexity of the patients. In fact, it is more reasonable to consider allocating the resources based on the complexity of the medical cases than purely on the patients’ amount. In addition, the last column of Table 8 declares that the RTS status of KWC is DRS, indicating that the HKHA should reduce its medical resource inputs.

**Table 8:** Specific data and efficiency scores of clusters, 2013.

Cluster	DI_1_	DI_2_	UI_1_	DO_1_	DO_2_	DO_3_	UO_1_	Score	RTS
HKEC	7226	3031	82.7	1187.01	248.93	1598.69	21.8	0.839	IRS
HKWC	7349	3135	74.2	1137.18	132.56	1374.04	16.2	0.754	IRS
KCC	8899	3547	87.2	1663.34	206.21	1979.47	26.8	0.913	DRS
KEC	6484	2371	85.9	902.60	315.83	1930.08	23.7	1	CRS
KWC	14076	6587	83.1	2430.17	580.13	3667.01	23.7	0.954	DRS
NTEC	10096	4515	84.8	1856.25	309.58	2314.03	21.6	0.839	DRS
NTWC	8309	3967	88.4	2410.10	360.06	1923.74	22.0	1	CRS

**Fig 2:**
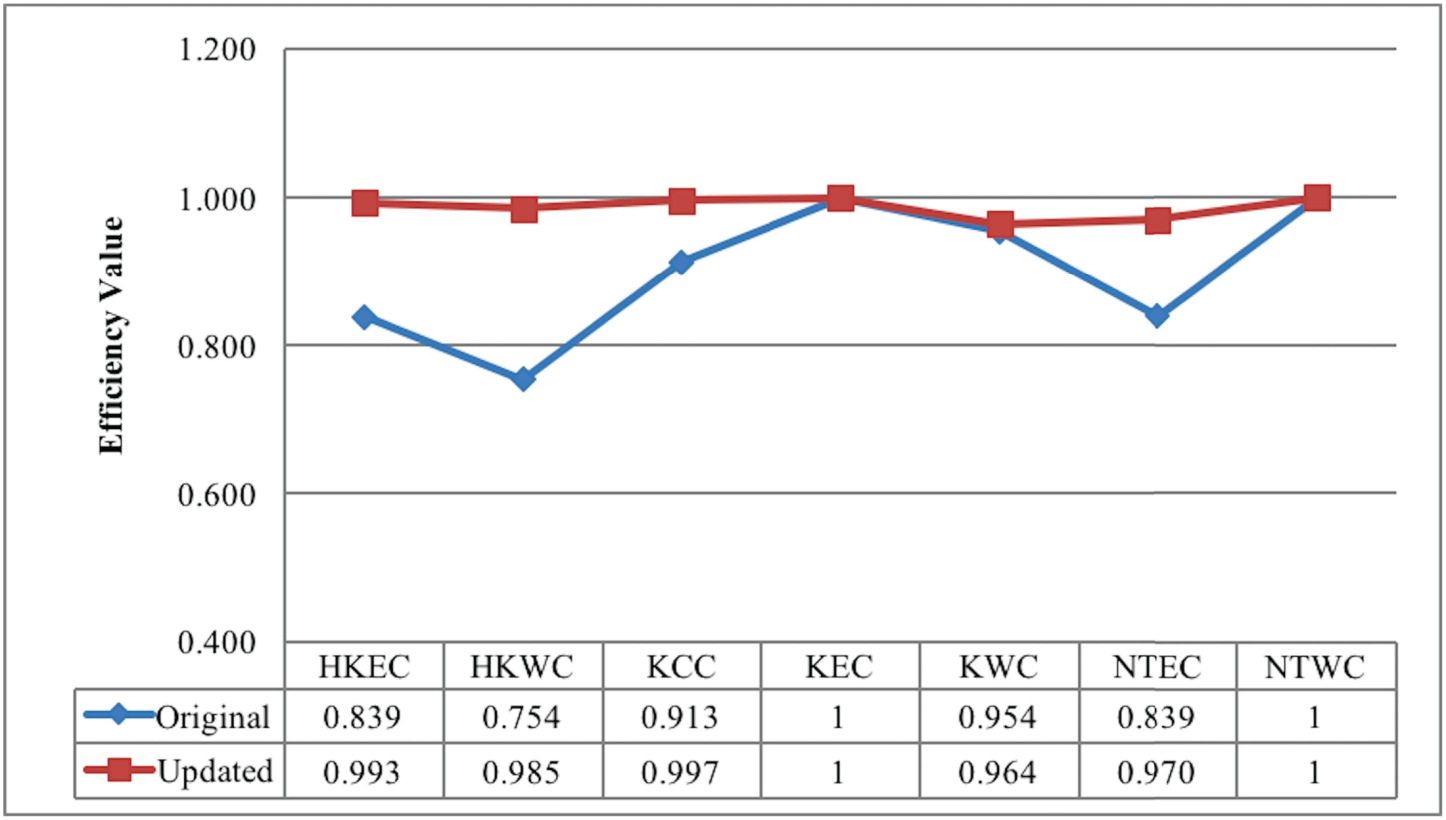
Original vs. Updated: Resource operation efficiencies of each cluster, 2013.

Table 9 displays the slacks of each cluster measured by GSBUP, which is able to further validate the idea mentioned above. It is observed that the ***s***^*D*−^ of HKEC and HKWC with IRS is almost equal to zero; while their ***s***^*G*+^ are not null, which means that the inefficient clusters with IRS are mainly caused by the inefficient resource utilization. On the other hand, the inefficient hospitals with DRS are mainly caused by the unfair resource allocation. This phenomenon can guide the HKHA policy makers to better control healthcare costs and improve healthcare efficiency under the service quality requirements. Considering all the slacks, the updated clusters’ efficiency scores based on GSBUP-LP model can be calculated, as shown in the last column of Table 9. The efficiency values of the comparison are displayed in Fig 2. It is observed that all the clusters’ efficiencies are improved significantly, which further demonstrates that it is necessary to change the ideas for the medical investment standard.

**Table 9:** Slacks of each cluster measured by GSBUP, 2013.

Cluster	$S_{1}^{D-}$	$S_{2}^{D-}$	$S_{1}^{I+}$	$S_{1}^{G+}$	$S_{2}^{G+}$	$S_{3}^{G+}$	$S_{1}^{B-}$	Score
HKEC	0.00	9.74	1.89	241.84	48.00	228.95	0.00	0.941
HKWC	0.00	9.06	1.52	508.21	72.97	276.84	0.00	0.972
KCC	984.85	392.57	0.25	274.36	146.76	0.00	4.21	1
KEC	0.00	0.00	0.00	0.00	0.00	0.00	0.00	1
KWC	1627.82	761.76	0.25	418.85	431.40	0.00	3.89	1
NTEC	1026.85	459.20	0.23	281.37	202.46	0.00	3.12	0.966
NTWC	0.00	0.00	0.00	0.00	0.00	0.00	0.00	1

#### Evaluation of malmquist productivity change index

Table 10 reports HKHA hospitals’ productivity change during the period 2000-2013. We observe that from 2000 to 2013, all the productivities of hospitals have made significant improvement. There are two major reasons: (i) SARS outbreak and (ii) Health services fees’ reformation. The first diagnosed patient with SARS was on 21 February 2003 and the disease had widely spread in HK within just two weeks. The peak of the epidemic started from 24 March 2003, and there was a rapid influx of infected residents in HA hospitals, particularly the United Christian Hospital which was soon overwhelmed. Therefore, its productivity improves by 17.87%, particularly, the TEC and FS improve by 4.77% and 13.76%, respectively (see Table 11). On 29 March 2003, the Government and HA decided to designate the Princess Margret Hospital, due to its specialty in treating infectious disease, to receive SARS patients after diverting all its existing patients to other hospitals. Thus, its FS only improves by 1.81%. However, since the government intensified a strong technical support to the Princess Margret Hospital for resisting SARS, its TEC improves by 9.87% during 2000-2003 (see Table 11). Besides, the speed of the patient flow increase was unexpected. The additional manpower from other hospitals was deployed to Princess Margret Hospital for help, which is to reduce the medical resources input equivalently for the other hospitals. With the contribution from other hospitals to share the workload, the designation of Princess Margret Hospital officially ended on 11 April 2003. Furthermore, with the assistance of the necessary operational and information technology systems, a revised public hospital fees structure was implemented at the beginning of 2003 together with an enhanced waiver mechanism to better provide the available public resources to those in need. Along with the soaring SARS attendances, there is improvement in TEC for almost all the hospitals.

**Table 10:** Malmquist productivity index of HKHA.

Hospital	2000-2003	2003-2007	2007-2010	2010-2013	Average (2007-2013)
Pamela Youde Nethersole Eastern	0.9774	1.0860	1.0013	0.9751	0.9882
Ruttonjee and Tang Shiu Kin	0.9699	1.0599	0.9854	0.9594	0.9724
St. John	0.9448	1.1717	1.0056	1.0047	1.0052
Tung Wah Eastern	0.9823	1.1623	1.2609	1.1923	1.2266
Queen Mary	0.9309	1.0407	0.9642	0.9205	0.9424
Tung Wah	0.9653	1.0584	0.9692	0.9784	0.9738
Queen Elizabeth	0.8547	1.0681	0.9322	0.9233	0.9278
Tseung Kwan O	0.9844	1.0174	1.0079	0.9615	0.9847
United Christian	0.8213	1.0647	0.9217	1.0015	0.9616
Caritas Medical Centre	0.9895	1.1140	0.9814	0.9660	0.9737
Kwong Wah	0.9909	1.1204	0.9722	1.0079	0.9901
Princess Margaret	0.8850	1.0142	0.9791	0.9148	0.9470
Yan Chai	0.9557	1.0163	0.9885	0.9789	0.9837
Alice Ho Miu Ling Nethersole	0.9889	1.1361	0.9629	0.9877	0.9753
North District	0.9890	1.1463	1.1594	1.0382	1.0988
Prince of Wales	0.8414	1.0602	0.8986	0.9971	0.9479
Pok Oi	0.9394	1.0468	0.5652	0.8925	0.7283
Tuen Mun	0.9666	1.0372	0.9710	0.8522	0.9116

**Table 11:** Factoring of malmquist productivity index of HKHA.

Hospital	2000-2003		Average (2007-2013)	
	TEC	FS	TEC	FS
Pamela Youde Nethersole Eastern	1.0004	0.9770	1.0000	0.9882
Ruttonjee and Tang Shiu Kin	0.9973	0.9725	1.0000	0.9724
St. John	1.0001	0.9447	0.9893	1.0160
Tung Wah Eastern	1.0224	0.9608	1.0184	1.2044
Queen Mary	0.9987	0.9321	0.9791	0.9625
Tung Wah	1.0000	0.9653	1.0001	0.9737
Queen Elizabeth	0.9787	0.8733	0.9981	0.9295
Tseung Kwan O	1.0000	0.9844	0.9999	0.9848
United Christian	0.9523	0.8624	0.9882	0.9731
Caritas Medical Centre	1.0001	0.9894	1.0001	0.9736
Kwong Wah	0.9931	0.9978	1.0013	0.9888
Princess Margaret	0.9013	0.9819	0.9747	0.9715
Yan Chai	0.9891	0.9662	1.0000	0.9837
Alice Ho Miu Ling Nethersole	1.0000	0.9889	0.9817	0.9935
North District	1.0031	0.9859	1.0134	1.0843
Prince of Wales	0.9723	0.8654	0.9681	0.9791
Pok Oi	1.0000	0.9394	1.0000	0.7289
Tuen Mun	0.9813	0.9443	0.9856	0.9249

Not surprisingly, the HKHA hospital productivity regresses from 2003 to 2007, since SARS has been gradually defeated during this period, and the patient attendances decrease a lot. In the periods, 2007-2010 and 2010-2013, the HKHA average productivity has generally increased except St. John Hospital, Tung Wah Eastern Hospital and North District Hospital, which are not the core and mainstream hospitals in HK. In particular, the St. John Hospital provides health services for the citizens living on the Cheung Chau Island, which is a geographically isolated place. Tung Wah Eastern Hospital and North District Hospital were once voted as the one with the highest emergency surgery mortality rates, therefore, their average TEC declines by 1.84% and 1.34% during 2007-2013 (see Table 11). In addition to the observations stated above, it is indicated in Table 10 that the productivity and FS of Pok Oi Hospital improve a lot during 2007-2013, as it has provided ED services since 2010.

## Relationship between exogenous factors and DEA efficiency scores

In Phase I, we have measured the HKHA efficiency changes from 2010 to 2013 by applying the GSBUP-Malmquist index. In Phase II, we will further explore the impact of some exogenours factors on HKHA efficiency by Tobit regression model.

### Exogenous factors selection

The HK healthcare system is composed of two sectors: a private track and a government sponsored public track. Citizens can access the public healthcare system at facilities operated by the HA. Insurance is not necessary for the eligible. However, the public system is geared toward emergency care, which means that if an attending nurse deems a case to be not critical, patients may sometimes spend hours waiting to see a doctor. Thus, the citizens with good financial condition sometimes prefer to choose the private hospitals for their efficient and high quality services. Private healthcare, however, generally is more expensive than the public healthcare system which mainly comes from the out-of-pocket household payment and commercial medical insurance pay-out.

According to the World Factbook in 2013, the Gini index of HK for households rose from 0.429 in 1976 to 0.557 in 2013, while that for economically active individuals increased from 0.411 in 1976 to 0.497 in 2013. The rising Gini index of both household and individual income are often regarded as the evidence of income inequality. Therefore, household income inequality has risen naturally because of the population composition change (e.g., more non-working elderly or single-parent household than those in the past) [40]. It is well known that HK is one of the most densely populated areas in the world, with an overall density of around 6300 people per square kilometer. At the same time, HK has one of the world’s lowest birth rates—1.11 per woman of child-bearing age in 2013, far below the replacement rate of 2.1. It is estimated that 26.8% of the population will age over 65 in 2033, rising from 12.1% in 2005.

Therefore, we consider from the aspects of economy, geography, education and demography to further explore the influence of exogenous factors. Table 12 provides the details of these factors, which are collected from the Hong Kong Census and Statistics Department [41].

**Table 12:** Exogenous factors and relevant symbols.

Exogenous factor	Symbol	Definition
Economy	Inc	Median monthly domestic household income
	PoDa	Poverty situation of persons with disabilities
Demography	PD	Population density
	PA65	Proportion of population aged 65 and over
Geography	LA	Land area
	POD/C	Proportion of patients come from the other districts (clusters)
Education	PE	Proportion of non-student population aged 20 and over having attained post-secondary education

### Measurement of Tobit regression model and its analysis results

The most widely used method to model the DEA scores against exogenous factors is Tobit regression, which is suitable when the dependent variables are either censored or corner solution outcomes [33, 42, 43]. It is validated in [43] that the Tobit regression model is powerful in representing the second stage in DEA models. The relationship between exogenous factors and cluster efficiencies can be described by the following model:

$$\begin{array}{ccc} {\text{HACE}}_{it} & = & \beta_{0}+\beta_{1}{\text{Inc}}_{it}+\beta_{2}{\text{PoDa}}_{it}+\beta_{3}{\text{PD}}_{it}+\beta_{4}{\text{PA65}}_{it}+\beta_{5}{\text{LA}}_{it}+\\ & & \beta_{6}{\text{POD/C}}_{it}+\beta_{7}{\text{PE}}_{it}+\epsilon_{it} \end{array}$$ (7)

where HACE_*it*_ describes the *i*th cluster’s efficiency in the *t*th year. The symbol denotes the corresponding exogenous factor’s value in the *i*th cluster of *t*th year. (*β*_0_, *β*_1_, …, *β*_7_) are the unknown coefficients, and *ϵ*_*it*_ ∼ *N*(0, *σ*) are the independent and identically normally distributed residuals of the observations. In this study, we use the maximum likelihood estimation toolbox in R [44] to obtain the Tobit regression results, as presented in Table 13.

**Table 13:** Tobit regression results.

Variable	Coefficient	Std. Error	z-Statistic	Prob.
Inc	-4.19E-05	1.62E-05	-2.5934	0.0095
PoDa	5.37E-04	1.94E-03	2.3158	0.0000
PD	0.012953	0.008213	1.5770	0.0413
PA65	-1.69E-07	5.46E-08	3.1025	0.0019
LA	1.72E-04	1.66E-04	1.0317	0.3022
POD/C	0.003148	0.001088	-2.8929	0.0037
PE	-0.00691	0.001484	-4.6541	0.0000
_CONS	1.004891	0.025887	3.8182	0.0000
**Adjusted R-squared**	0.706913			
**Log likelihood**	15.52829			

Firstly, the poverty situation of persons with disabilities has a positive influence on HA hospital efficiency; while median monthly domestic household income and education level affect the environmental efficiency negatively. Since the median income of each cluster has almost no relationship with population density (i.e., correlation coefficient = 0.03), the empirical results reveal an interesting phenomenon: the public hospitals that serve in a richer district, has a relatively lower efficiency in HK, since people with higher degree of education and income prefer to accepting higher quality service from the private hospitals.

Secondly, the proportion of population aged over 65 and HA hospital efficiency has a negative correlation, because the elderly are more likely to suffer from life-threatening diseases. Many elderly patients are physically too weak to see the doctors. Moreover, one out of three elderly HK residents lives under poverty or disabilities, so they are usually unable to take good care of themselves once in sick. In other words, the aged population might increase the complications and the mortality rate. Therefore, the aged population have a negative correlation with the HA hospital efficiency.

Thirdly, the proportion of patients come from the other clusters takes a positive correlation with the efficiency. The HA hospitals in HK are divided into seven clusters according to their geographical locations so that the healthcare service is evenly distributed across HK. Although the patients are expected to receive the same level of medical care, wherever they abide in, the quality of medical care are various across different locations. Thus, some patients abiding in the relative poverty districts may try to receive the better treatments from the other districts’ hospitals, such as Queen Mary Hospital, belonging to the HKWC. These types of patients will increase the workload of some hospitals, which is validated by the regression results.

Finally, the land area has no significant influence on HA hospital efficiency while population density has a weak positive influence. For example, New Territories has the largest land area in HK, while its population density is the lowest. Kowloon has the highest population density, at the same time, most of the poor with disabilities abide in this cluster. In addition, the land area and population density of Hong Kong Island lie in the middle, but the citizens are relatively richer. In conclusion, the geography factor has no significant influence on HA hospital efficiency.

## Conclusion

In DEA model, efficiency score is closely related to the quantities of inputs and outputs. An ideal scenario with the highest efficiency in a healthcare setting would be treating the most patients using the least healthcare expenditure. However, the reception of the patients beyond the hospital capacity will generate a congestion problem, even resulting in some unexpected mortality, where a high patient mortality rate not only reflects a poor medical quality, but also may cause a dispute. Thus, it is regarded as an UO and is expected to be as low as possible. On the other hand, the in-patient discharge rate is a powerful indicator to represent whether this hospital could provide a good quality of service, which is expected to be as high as possible, as an UI. Moreover, the measurement of hospital efficiency might be biased if the undesirable factors are ignored.

By integrating the GSBUP-DEA model and Malmquist index, this paper examines the resource efficiency of HKHA based on the panel data from 2000 to 2013. Then, through the Tobit regression model, some exogenous factors’ influences are tested. The empirical results show that the HKHA hospital efficiencies of each hospital and cluster are significant different. In general, the healthcare efficiency of hospitals belonging to the KEC and NTWC are significantly higher than the other hospitals, which has a close correlation with demographic composition, geographic condition and economic position. By analyzing the evaluation of productivity change over time, we observed that the SARS outbreak has a strong impact on the hospital efficiency. In addition, we found an interesting phenomenon that the public hospital serving in a richer district with high population density has a relatively lower efficiency, as HK people with better economic condition prefer to accept higher quality service from the private hospital.

In recent decades, HKHA has been making efforts to improve the healthcare services, but there still exist some problems like unfair resource allocation and poor management, as reported by the Hong Kong medical legislative committee. One critical consequence of these problems is low healthcare efficiency of hospitals, leading to low satisfaction among patients. In conclusion, we provide two suggestions on how to improve the HKHA hospital efficiency in the future: (i) according to the original resource allocation system, the HKHA policy makers need to properly increase the input for the inefficient hospitals with IRS, such as Tung Wah Eastern Hospital and save some investment for the inefficient hospitals with DRS, such as Tung Wah Eastern Hospital Prince of Wales Hospital. (ii) It is necessary to change the medical investment standard. It may be better to consider patient attendance and hospital current efficiency simultaneously. Such a new system can solve the problem of unfair resource allocation effectively.

In the future, we will consider the patient service quality and use IDEA (Imprecise data) models to help HKHA policy makers find the technology backward hospitals. To further improve the efficiency in resource utilization and meet the need of growing healthcare demand due to aging population, the internal resource allocation system should be personalized and modernized at an individual level by involving heterogeneity of patients need. Thus, we will also attempt to investigate the simulation methods for resource optimization by integrating heterogeneous factors at individual level in our future works.

## A The linear programming of GSBUP

By applying the Charnes-Cooper transformation, [GSBUP] can be transformed into a linear programming.

Suppose *t* (*t* > 0) is a scalar variable, let:

$$\begin{array}{cc} & t=\frac{1}{\sum_{l=1}^{q}\sigma_{l}\gamma_{l}+\sum_{r=1}^{s}\mu_{r}\beta_{r}}\\ t\alpha_{i}=a_{i}\;t\gamma_{l}=b_{l} & \;t\beta_{r}=c_{r}\;t\theta_{h}=d_{h}\;t\lambda_{j}=\zeta_{j}\;\left(\forall j,i,l,r,h\right) \end{array}$$

Since

$$\begin{array}{c} \sum_{l=1}^{q}\sigma_{l}b_{l}+\sum_{r=1}^{s}\mu_{r}c_{r}=t\left(\sum_{l=1}^{q}\sigma_{l}\gamma_{l}+\sum_{r=1}^{s}\mu_{r}\beta_{r}\right)=\frac{\sum_{l=1}^{q}\sigma_{l}\gamma_{l}+\sum_{r=1}^{s}\mu_{r}\beta_{r}}{\sum_{l=1}^{q}\sigma_{l}\gamma_{l}+\sum_{r=1}^{s}\mu_{r}\beta_{r}}=1 \end{array}$$

the linear programming of [GSBUP] can be transformed as follows:

**[GSBUP-LP]**

$$\begin{array}{cc} & \min\;\;\tau_{0}=\sum_{i=1}^{m}\omega_{i}a_{i}+\sum_{h=1}^{k}\nu_{h}d_{h}\\ & s.t.\;\left\{ \begin{array}{c} \sum_{l=1}^{q}\sigma_{l}b_{l}+\sum_{r=1}^{s}\mu_{r}c_{r}=1\\ \sum_{j=1}^{n}x_{ij}^{D}\zeta_{j}=a_{i}x_{i0}^{D}\;\left(i=1,2,\ldots ,m\right)\\ \sum_{j=1}^{n}x_{lj}^{I}\zeta_{j}=b_{l}x_{l0}^{I}\;\left(l=1,2,\ldots ,q\right)\\ \sum_{j=1}^{n}y_{rj}^{G}\zeta_{j}=c_{r}y_{r0}^{G}\;\left(r=1,2,\ldots ,s\right)\\ \sum_{j=1}^{n}y_{hj}^{B}\zeta_{j}=d_{h}y_{h0}^{B}\;\left(h=1,2,\ldots ,k\right)\\ \sum_{j=1}^{n}\zeta_{j}=t\\ \zeta_{j}\geq 0,\;0\leq a_{i}\leq t,\;b_{l}\geq t,\;c_{r}\geq t,\;0\leq d_{h}\leq t\;t>0\;\left(\forall j,i,l,r,h\right) \end{array}\right. \end{array}$$ (8)

## B Proof of Theorem 1

Based on Eq (4), ${\hat{\text{DMU}}}_{0}$ can be expressed as:

$$\begin{array}{cc} & \min\;\;{\hat{\delta }}_{0}=\frac{\sum_{i=1}^{m}\omega_{i}{\hat{\alpha }}_{i}+\sum_{h=1}^{k}\nu_{h}{\hat{\theta }}_{h}}{\sum_{l=1}^{q}\sigma_{l}{\hat{\gamma }}_{l}+\sum_{r=1}^{s}\mu_{r}{\hat{\beta }}_{r}}\\ & s.t.\;\left\{ \begin{array}{c} \sum_{j=1}^{n}x_{ij}^{D}{\hat{\lambda }}_{j}+{\hat{x}}_{i0}^{D}{\hat{\lambda }}_{n+1}={\hat{\alpha }}_{i}{\hat{x}}_{i0}^{D}\;\left(i=1,2,\ldots ,m\right)\\ \sum_{j=1}^{n}x_{lj}^{I}{\hat{\lambda }}_{j}+{\hat{x}}_{l0}^{I}{\hat{\lambda }}_{n+1}={\hat{\gamma }}_{l}{\hat{x}}_{l0}^{I}\;\left(l=1,2,\ldots ,q\right)\\ \sum_{j=1}^{n}y_{rj}^{G}{\hat{\lambda }}_{j}+{\hat{y}}_{r0}^{G}{\hat{\lambda }}_{n+1}={\hat{\beta }}_{r}{\hat{y}}_{r0}^{G}\;\left(r=1,2,\ldots ,s\right)\\ \sum_{j=1}^{n}y_{hj}^{B}{\hat{\lambda }}_{j}+{\hat{y}}_{h0}^{B}{\hat{\lambda }}_{n+1}={\hat{\theta }}_{h}{\hat{y}}_{h0}^{B}\;\left(h=1,2,\ldots ,k\right)\\ \sum_{j=1}^{n}{\hat{\lambda }}_{j}+{\hat{\lambda }}_{n+1}=1\\ {\hat{\lambda }}_{j}\geq 0\;\left(j=1,2,\ldots ,n+1\right)\\ 0\leq {\hat{\alpha }}_{i}\leq 1,\;{\hat{\gamma }}_{l}\geq 1,\;{\hat{\beta }}_{r}\geq 1,\;0\leq {\hat{\theta }}_{h}\leq 1\;\left(\forall i,l,r,h\right) \end{array}\right. \end{array}$$ (9)

Suppose (${\hat{\lambda }}^{*},{\hat{\alpha }}^{*},{\hat{\beta }}^{*},{\hat{\gamma }}^{*},{\hat{\theta }}^{*}$) is an optimal solution of Eq (9). According to Definition 2, the constraints of Eq (9) can be transformed as follows:

$$\begin{array}{cc} & \sum_{j=1}^{n}x_{ij}^{D}{\hat{\lambda }}_{j}^{*}+{\hat{x}}_{i0}^{D}{\hat{\lambda }}_{n+1}^{*}=\sum_{j=1}^{n}x_{ij}^{D}{\hat{\lambda }}_{j}^{*}+\sum_{j=1}^{n}x_{ij}^{D}\lambda_{j}^{*}{\hat{\lambda }}_{n+1}^{*}=\sum_{j=1}^{n}\left({\hat{\lambda }}_{j}^{*}+{\hat{\lambda }}_{n+1}^{*}\lambda_{j}^{*}\right)x_{ij}^{D}={\hat{\alpha }}_{i}^{*}{\hat{x}}_{i0}^{D}={\hat{\alpha }}_{i}^{*}\alpha_{i}^{*}x_{i0}^{D}\;\left(\forall i\right)\\ & \sum_{j=1}^{n}x_{lj}^{I}{\hat{\lambda }}_{j}^{*}+{\hat{x}}_{l0}^{I}{\hat{\lambda }}_{n+1}^{*}=\sum_{j=1}^{n}x_{lj}^{I}{\hat{\lambda }}_{j}^{*}+\sum_{j=1}^{n}x_{lj}^{I}\lambda_{j}^{*}{\hat{\lambda }}_{n+1}^{*}=\sum_{j=1}^{n}\left({\hat{\lambda }}_{j}^{*}+{\hat{\lambda }}_{n+1}^{*}\lambda_{j}^{*}\right)x_{lj}^{I}={\hat{\gamma }}_{l}^{*}{\hat{x}}_{l0}^{I}={\hat{\gamma }}_{l}^{*}\gamma_{l}^{*}x_{l0}^{I}\;\left(\forall l\right)\\ & \sum_{j=1}^{n}y_{rj}^{G}{\hat{\lambda }}_{j}^{*}+{\hat{y}}_{r0}^{G}{\hat{\lambda }}_{n+1}^{*}=\sum_{j=1}^{n}y_{rj}^{G}{\hat{\lambda }}_{j}^{*}+\sum_{j=1}^{n}y_{rj}^{G}\lambda_{j}^{*}{\hat{\lambda }}_{n+1}^{*}=\sum_{j=1}^{n}\left({\hat{\lambda }}_{j}^{*}+{\hat{\lambda }}_{n+1}^{*}\lambda_{j}^{*}\right)y_{rj}^{G}={\hat{\beta }}_{r}^{*}{\hat{y}}_{r0}^{G}={\hat{\beta }}_{r}^{*}\beta_{r}^{*}y_{r0}^{G}\;\left(\forall r\right)\\ & \sum_{j=1}^{n}y_{hj}^{B}{\hat{\lambda }}_{j}^{*}+{\hat{y}}_{h0}^{B}{\hat{\lambda }}_{n+1}^{*}=\sum_{j=1}^{n}y_{hj}^{B}{\hat{\lambda }}_{j}^{*}+\sum_{j=1}^{n}y_{hj}^{B}\lambda_{j}^{*}{\hat{\lambda }}_{n+1}^{*}=\sum_{j=1}^{n}\left({\hat{\lambda }}_{j}^{*}+{\hat{\lambda }}_{n+1}^{*}\lambda_{j}^{*}\right)y_{hj}^{B}={\hat{\theta }}_{h}^{*}{\hat{y}}_{h0}^{B}={\hat{\theta }}_{h}^{*}\theta_{h}^{*}y_{h0}^{B}\;\left(\forall h\right) \end{array}$$

$$\begin{array}{cc} & \sum_{j=1}^{n}{\hat{\lambda }}_{j}^{*}+{\hat{\lambda }}_{n+1}^{*}=\sum_{j=1}^{n}{\hat{\lambda }}_{j}^{*}+{\hat{\lambda }}_{n+1}^{*}\sum_{j=1}^{n}\lambda_{j}^{*}=\sum_{j=1}^{n}\left({\hat{\lambda }}_{j}^{*}+{\hat{\lambda }}_{n+1}^{*}\lambda_{j}^{*}\right)=1\;\left(\forall j\right) \end{array}$$

Because we have known that $0\leq {\hat{\alpha }}_{i}^{*}\alpha_{i}^{*}\leq 1$, ${\hat{\gamma }}_{l}^{*}\gamma_{l}^{*}\geq 1$, ${\hat{\beta }}_{r}^{*}\beta_{r}^{*}\geq 1$, and $0\leq {\hat{\theta }}_{h}^{*}\theta_{h}^{*}\leq 1$ (∀*i*, *l*, *r*, *h*). Thus, (${\hat{\lambda }}^{*}+{\hat{\lambda }}_{n+1}^{*}\lambda^{*},{\hat{\alpha }}^{*}\alpha^{*},{\hat{\beta }}^{*}\beta^{*},{\hat{\gamma }}^{*}\gamma^{*},{\hat{\theta }}^{*}\theta^{*}$) is a feasible solution of Eq (4), and then we can get an inequality function as follows:

$$\begin{array}{c} \frac{\sum_{i=1}^{m}\omega_{i}{\hat{\alpha }}_{i}^{*}\alpha_{i}^{*}+\sum_{h=1}^{k}\nu_{h}{\hat{\theta }}_{h}^{*}\theta_{h}^{*}}{\sum_{l=1}^{q}\sigma_{l}{\hat{\gamma }}_{l}^{*}\gamma_{l}^{*}+\sum_{r=1}^{s}\mu_{r}{\hat{\beta }}_{r}^{*}\beta_{r}^{*}}\geq \frac{\sum_{i=1}^{m}\omega_{i}\alpha_{i}^{*}+\sum_{h=1}^{k}\nu_{h}\theta_{h}^{*}}{\sum_{l=1}^{q}\sigma_{l}\gamma_{l}^{*}+\sum_{r=1}^{s}\mu_{r}\beta_{r}^{*}} \end{array}$$

Because $0\leq {\hat{\alpha }}_{i}^{*}\leq 1$, ${\hat{\gamma }}_{l}^{*}\geq 1$, ${\hat{\beta }}_{r}^{*}\geq 1$, and $0\leq {\hat{\theta }}_{h}^{*}\leq 1$ (∀*i*, *l*, *r*, *h*), in order to satisfy the inequality Eq (10), there must be (α*, β*, γ*, θ*) = (**1**, **1**, **1**, **1**). Thus, we can demonstrate that the projection, ${\hat{\text{DMU}}}_{0}=\left({\hat{x}}_{0}^{D},{\hat{y}}_{0}^{G},{\hat{x}}_{0}^{I},{\hat{y}}_{0}^{B}\right)$ is GSBUP-efficient, according to Definition 1.

## C The malmquist index

$$\begin{array}{cc} \delta_{0}^{t}\left(x_{0}^{D,t},y_{0}^{G,t},x_{0}^{I,t},y_{0}^{B,t}\right) & =\min\;\;\delta_{0}=\frac{\sum_{i=1}^{m}\omega_{i}\alpha_{i}+\sum_{h=1}^{k}\nu_{h}\theta_{h}}{\sum_{l=1}^{q}\sigma_{l}\gamma_{l}+\sum_{r=1}^{s}\mu_{r}\beta_{r}}\\ s.t.\; & \left\{ \begin{array}{c} \sum_{j=1}^{n}x_{ij}^{D,t}\lambda_{j}=\alpha_{i}x_{i0}^{D,t}\;\left(i=1,2,\ldots ,m\right)\\ \sum_{j=1}^{n}x_{lj}^{I,t}\lambda_{j}=\gamma_{l}x_{l0}^{I,t}\;\left(l=1,2,\ldots ,q\right)\\ \sum_{j=1}^{n}y_{rj}^{G,t}\lambda_{j}=\beta_{r}y_{r0}^{G,t}\;\left(r=1,2,\ldots ,s\right)\\ \sum_{j=1}^{n}y_{hj}^{B,t}\lambda_{j}=\theta_{h}y_{h0}^{B,t}\;\left(h=1,2,\ldots ,k\right)\\ \sum_{j=1}^{n}\lambda_{j}\in \Gamma \\ \lambda_{j}\geq 0,\;0\leq \alpha_{i}\leq 1,\;\gamma_{l}\geq 1,\;\beta_{r}\geq 1,\;0\leq \theta_{h}\leq 1\;\left(\forall j,i,l,r,h\right) \end{array}\right. \end{array}$$ (10)

$$\begin{array}{cc} \delta_{0}^{t}\left(x_{0}^{D,t+1},y_{0}^{G,t+1},x_{0}^{I,t+1},y_{0}^{B,t+1}\right) & =\min\;\;\delta_{0}=\frac{\sum_{i=1}^{m}\omega_{i}\alpha_{i}+\sum_{h=1}^{k}\nu_{h}\theta_{h}}{\sum_{l=1}^{q}\sigma_{l}\gamma_{l}+\sum_{r=1}^{s}\mu_{r}\beta_{r}}\\ s.t.\; & \left\{ \begin{array}{c} \sum_{j=1}^{n}x_{ij}^{D,t}\lambda_{j}=\alpha_{i}x_{i0}^{D,t+1}\;\left(i=1,2,\ldots ,m\right)\\ \sum_{j=1}^{n}x_{lj}^{I,t}\lambda_{j}=\gamma_{l}x_{l0}^{I,t+1}\;\left(l=1,2,\ldots ,q\right)\\ \sum_{j=1}^{n}y_{rj}^{G,t}\lambda_{j}=\beta_{r}y_{r0}^{G,t+1}\;\left(r=1,2,\ldots ,s\right)\\ \sum_{j=1}^{n}y_{hj}^{B,t}\lambda_{j}=\theta_{h}y_{h0}^{B,t+1}\;\left(h=1,2,\ldots ,k\right)\\ \sum_{j=1}^{n}\lambda_{j}\in \Gamma \\ \lambda_{j}\geq 0,\;0\leq \alpha_{i}\leq 1,\;\gamma_{l}\geq 1,\;\beta_{r}\geq 1,\;0\leq \theta_{h}\leq 1\;\left(\forall j,i,l,r,h\right) \end{array}\right. \end{array}$$ (11)

In a similar way, using *t* + 1 instead of *t* for the Models (10) and (11), we can get $\delta_{0}^{t+1}\left(x_{0}^{D,t+1},y_{0}^{G,t+1},x_{0}^{I,t+1},y_{0}^{B,t+1}\right)$ and $\delta_{0}^{t+1}\left(x_{0}^{D,t},y_{0}^{G,t},x_{0}^{I,t},y_{0}^{B,t}\right)$.

## Supporting information

S1 File HKHA real data for experiment. (XLSX)
