# Correction: Hong Kong Hospital Authority resource efficiency evaluation: Via a novel DEA-Malmquist model and Tobit regression model

**DOI:** 10.1371/journal.pone.0193266

**Published:** 2018-02-15

**Authors:** Hainan Guo, Yang Zhao, Tie Niu, Kwok-Leung Tsui

The Data Availability statement for this paper is incorrect. The correct statement is: Data from the Hospital Authority Statistical Report HKSAR are publicly available and can be found in the Supporting Information files or at the following URL: http://www.ha.org.hk/visitor/ha_index.asp?Lang=CHIB5.
